# An Impact Assessment of Par-Baking and Storage on the Quality of Wheat, Whole Wheat, and Whole Rye Breads

**DOI:** 10.3390/foods13020224

**Published:** 2024-01-10

**Authors:** Celeste Verbeke, Els Debonne, Hannah Van Leirsberghe, Filip Van Bockstaele, Mia Eeckhout

**Affiliations:** 1 Ghent University, Faculty of Bioscience Engineering, Department of Food Technology, Safety and Health, Research Unit Cereal and Feed Technology, Valentin Vaerwyckweg 1, 9000 Ghent, Belgium; celeste.verbeke@ugent.be (C.V.); els.debonne@ugent.be (E.D.); hannah.vanleirsberghe@ugent.be (H.V.L.); 2 Ghent University, Faculty of Bioscience Engineering, Department of Food Technology, Safety and Health, Food Structure and Function Research Group, Coupure Links 653, 9000 Ghent, Belgium; filip.vanbockstaele@ugent.be

**Keywords:** par-baking, texture, whole meal, wheat, rye, bread quality

## Abstract

Par-baking technology increases the production efficiency of bread. However, the degree of par-baking can vary significantly amongst product types and intended sales markets, leading to substantial differences in the quality attributes of the finished product. The objective of this study was to explore the impact of the degree of par-baking on the technological quality of wheat, whole wheat, and whole rye bread (95, 75, and 50% of full baking time). More specifically, this study focused on the starch pasting behavior of different flour formulations, the crumb core temperature during par-baking, and the influence of the degree of par-baking on the bread characteristics of (composite) wheat bread as a function of storage time. The quality attributes of par-baked bread (0 and 4 days after par-baking) and fully baked bread (0 and 2 days after full baking) were assessed. A reduction in the degree of par-baking from 95 to 50% resulted over time in 19.4% less hardening and 8.6% more cohesiveness for the re-baked wheat breads. Nevertheless, it also negatively impacted springiness (−9.1%) and adhesion (+475%). It is concluded that using the core temperature to define the degree of par-baking is not sufficient for bread loaves intended to be consumed over time, but the results indicate that reducing the degree of par-baking can be beneficial for certain quality aspects of the breads.

## 1. Introduction

Throughout Europe, the Middle East, and Africa, the Compound Annual Growth Rate (CAGR) forecast of the par-baked bread market is 4.3% between 2023 and 2032. The growth of this segment is due to the growth of industrial bakeries in Europe and the increasing consumption of bakery products [1]. Par-baked breads can either be stored under frozen storage (F-PB), under chilled or ambient temperatures, or in a modified atmosphere (MAP) [2]. One of the major advantages of par-baking is the flexibility provided for bakers, restaurants, and consumers, who can easily bake off products according to demand [2,3,4]. Although frozen storage is currently the most important strategy for par-baked breads due to its lower packaging demand and better shelf-life stability, freezing can speed up the retrogradation of bread after full baking, thereby shortening the physico-chemical shelf life of bread [5]. Bosmans et al. [6] stated that a lower storage temperature leads to a more rigid starch network and higher bread crumb firmness. Furthermore, they found that shorter baking times result in less retrogradation during storage, a less extended starch network, and smaller changes in crumb firmness and elasticity [6].

Par-baked bread intended for B2B purposes is often 95% par-baked. However, the degree of par-baking (PB) can vary from 50 to 95% depending on the type of product and the sales market [7,8]. In the literature, several authors report on the degree of par-baking as a factor influencing bread quality [7,8,9,10,11]. Fik and Surwka [8] found that the optimal time for the par-baking of F-PB bread is situated in the range from 74 to 86% of the time that is needed for the conventional baking process. Almeida and Chang [7] investigated the effect of the pre-baking time and crumb core temperature on structural changes in the dough of French bread made with whole wheat flour. They found that the starch gelatinization temperature (76 °C) was reached after 68.6% of the full baking time, whereas a core temperature of 93 °C was reached after 88.5% of the pre-baking time [7]. Furthermore, the starch gelatinization process was not finalized until the end of the pre-baking step but continued during the re-baking process. The temperature of gelatinization is dependent on the amylopectin crystal stability [12]. The baking process can also be mathematically modeled, following some general assumptions, such as (1) that bread is homogeneous and porous, (2) Fourier’s Law of heat by conduction, (3) that liquid diffusion occurs only in the crumb and vapor diffusion in the crust, and (4) that no volume change occurs [13,14]. Although in the literature, reports on the degree of PB in combination with the bread core temperature are found [7,15], it is still under investigation what the actual implication of the PB degree and crumb core temperature is on the end-product’s quality. In a study by Debonne et al. [16], par-baked wheat and rye breads from a small-scale industrial bakery reached crumb core temperatures of 93 °C and 97 °C, respectively. Full baking of previously PB breads to a core temperature of 65 °C was required for obtaining a good end-product quality. In conventional bread baking, the optimal baking time is defined by a critical time for starch gelatinization and a quality time, i.e., the time necessary to achieve a target value for a given quality attribute [17]. However, within the PB technology, the starch gelatinization procedure can continue in the full baking step [7], and the concept of optimal baking time or grade must be revised.

Moreover, the industry would benefit from gaining more insight into the interaction between the baking process parameters and end-product quality during its shelf life. This is of importance, as bread is one of the most wasted foods [18]. In the Flemish region of Belgium alone, 25% of the 270 million kg bakery products produced annually are wasted [19]. Jung et al. [20] summarized other values from the literature. The percentages of wasted bread in Sweden, Portugal, Norway, Finland, the Netherlands, New Zealand, and Saudi Arabia, are, respectively, 12–17, 8, 27, 13, 22, 23, and 19%. This was supplemented with absolute values for the United Kingdom (292 million kg) and Sweden (80 million kg).

The aim of this study was to investigate the impact of the degree of par-baking on different types of bread, including wheat, whole wheat, and rye bread. Furthermore, the lack of consensus found in the literature between par-baking based on the crumb core temperature and the percentage of the full baking time led to this study. Clarity was found by studying the influence of flour composition on starch pasting behavior and crumb core temperature and the influence of the degree of par-baking on the bread characteristics of par-baked and fully baked bread as a function of time.

## 2. Materials and Methods

### 2.1. Materials

*Epi B* wheat flour (WF) was purchased from Paniflower (Merksem, Belgium). *Integraal Puur* whole wheat (WWM) and *Rogge St Vith* whole rye meal (WRM) were kindly contributed by Ceres (Brussels, Belgium). The following characteristics were provided by the manufacturers: max. 15.5 g water/100 g flour and 12–13 g protein/100 g dm. The ash content was 0.5 g, 1.2 g, and 1.4 g/100 g, respectively, for WF, WWM, and WRM. Other ingredients such as dried yeast (*S. cerevisiae*, Algist Bruggeman) and refined table salt were obtained from the local supermarket.

### 2.2. Starch Pasting Properties

The starch pasting properties of WF, WWM, and WRM, as well as 50:50 blends of WWM:WF and WRM:WF, were assessed using a Rheometer MCR 102 with starch cell (Anton Paar GmbH, Graz, Austria). The AACC International Method 76-21-02 [21] was adapted to obtain the following procedure [22]: a 2.8 g sample (adjusted for 14% moisture content) was dispersed in 20 mL of distilled water. In a pre-shear phase, the suspension was mixed at 960 rpm and heated to 50 °C. During the pasting test, the rotation speed was maintained at 160 rpm. Initially, the temperature was held at 50 °C for 1 min, then raised to 95 °C at a constant rate of 5 °C/min, and maintained at that temperature for 5 min. Subsequently, the sample was cooled back down to 50 °C at 5 °C/min, and finally, held constant at 50 °C for 2 min.

The obtained parameters were initial viscosity, pasting temperature, peak viscosity, peak temperature, holding strength, breakdown, final viscosity, setback from peak, and total setback.

### 2.3. Bread Making Procedure

In this research study, three types of pan bread were prepared in-house. White bread (W) was made using 100% WF. For whole wheat (WW) and whole rye (WR) bread, a flour mixture consisting of 50% whole meal (either wheat or rye) and 50% white flour was applied. The dough composition included 1.5% table salt, 1% dry yeast, 0.1% malt flour, and 25 ppm ascorbic acid on flour weight basis. The optimal amount of water required for each specific formula was determined with the Brabender Farinograph to obtain optimal dough consistency of 500 BU. The acquired values are shown in Table 1. 

The ingredients were mixed and kneaded for 7 min using a De Danieli spiral mixer. The obtained dough was allowed to prove for 10 min at 30 °C and a relative humidity of 80–90% in a Panimatic (Souppes-sur-Loing, France) P16 proofing cabinet. Division into 400 g pieces was followed by mechanical rounding and a secondary fermentation of 30 min. After the degassing step, the dough was placed in greased baking pans for the final proof (65 min). The breads were baked in a Hein Bakelux convection oven (Strassen, Luxembourg). This study considered a baking program of 26 min, consisting of 11.5 min at 230 °C with 20 steam pulses, followed by 14.5 min at 200 °C, to result in a conventionally fully baked product. Based on this consideration, the loaves were par-baked for 50, 75, and 95% of the conventional baking time, that is, respectively, 13 min, 19.5 min, and 24.7 min. After baking, the breads were cooled at room temperature for 2 h before evaluation or packaging. The par-baked breads were individually packaged in sealed PA/PE-bags (90 µm thickness) and stored at room temperature (20 ± 1 °C). A full-bake step (3 min at 230 °C with 10 steam pulses, 6 min at 200 °C) followed after four days of storage, and after cooling, the breads were packaged in LDPE-bags (50 µm thickness). Each baking test was performed in duplicate.

### 2.4. Crumb Core Temperature

Crumb core temperatures were measured by K-type thermocouples (Ø 1.5 mm, Testo, Ternat, Belgium). The probes were positioned at a predetermined fixed height to ensure measurement at the center of the baked bread. Temperature readings were recorded every 10 s using a 175 T3-datalogger (Testo, Ternat, Belgium). Two replicates were conducted for each bread type.

### 2.5. Technological Bread Evaluation

Bread mass was measured using a KERN balance (±0.01 g), and bread volume (mL) was measured with a Volscan Profiler 600 (Stable Micro Systems, Godalming, UK). Bread crumb moisture content (MC) was determined using the AACC International Method 44-15.02 [23]. Crumb samples were taken from the middle section of the breads and were weighed before and after drying using an analytical balance (±0.0001 g). For the Texture Profile Analysis (TPA) a cylindrical plexiglass probe (Ø 25 mm, P/25P) made two compressions (40% strain, test speed 1.7 mm/s, 5 s pause) on three stacked slices of bread (9 mm each). By this analysis, hardness, springiness, cohesion, chewiness, and resilience were assessed. The CIELab color parameters of the crust were assessed after each baking phase using a CM700d/600d spectrophotometer (Konica Minolta, Tokyo, Japan), after standardization with a white calibration plate. L* expresses lightness and ranges from 0 to 100; a* and b* both range from −120 to 120 and represent, respectively, green(−) to red(+) and blue(−) to yellow(+) colors.

Four evaluation moments were considered for the breads: after the par-bake step (PB0), after four storage days of the par-baked breads (PB4), after the full-bake step (FB0), and after two storage days of the fully baked breads (FB2). Bread volume was measured at PB0; crust color at PB0 and FB0; and bread mass, MC, and TPA at PB0, PB4, FB0, and FB2.

### 2.6. Statistical Analysis

All experiments were at least performed in triplicate. Data analysis was carried out with SPSS Statistics (Version 27, IBM, Armonk, NY, USA). As the prerequisite of normality was not met, experimental data were submitted to a Kruskal–Wallis test with Dunn’s test to assess significant differences between groups (with Bonferroni correction). Principal component analysis (PCA) was conducted in R [24] on the MC, bread mass, hardness, adhesiveness, springiness, cohesiveness, chewiness, and resilience parameters. PCA was performed using the built-in R function prcomp. Graphic representation of the PCA was created using the factoextra- and ggplot2-packages [25,26]. Other graphs were rendered with SigmaPlot (Version 15, Inpixon, Palo Alto, CA, USA).

## 3. Results and Discussion

### 3.1. Starch Pasting Properties

**Figure 1:**
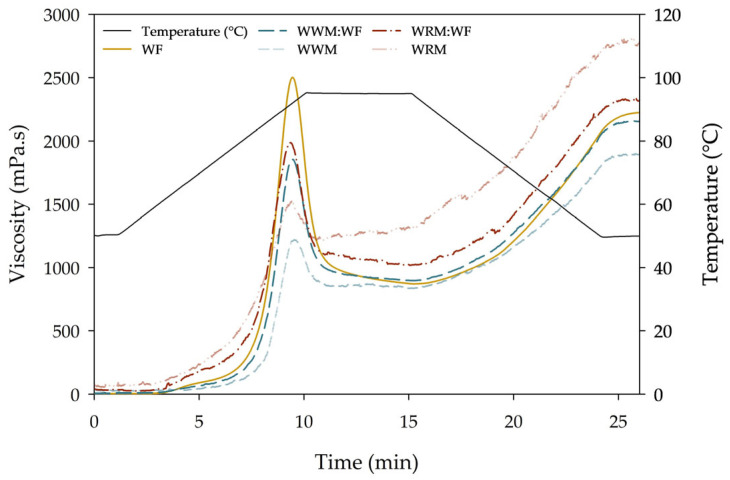
Average pasting behavior of wheat flour (WF), whole wheat meal (WWM), whole rye meal (WRM) (*n* = 6), and 50:50 blends consisting of WWM:WF and WRM:WF (*n* = 3).

By means of a rheometer and starch pasting cell, the heating and cooling process of baking was simulated in order to discover the potential effects of flour composition on the starch gelatinization process. Unlike in bread, there is an excess of water present during these rheological measurements. Figure 1 shows the pasting profiles of wheat flour (WF), whole wheat meal (WWM), whole rye meal (WRM), and 50:50 combinations of WWM:WF and WRM:WF. The data table is included in the Supplementary Materials (Table S1). The difference in pasting profiles indicates that the flour blends will behave differently during cooking and processing. The pasting temperatures varied from 57.6 ± 0.8 °C for WF to 73.1 ± 6.7 °C for WWM. The pasting temperatures did not significantly differ between WRM and WRM:WF (borderline significance, *p* = 0.05), indicating that from 50% of whole rye meal, the pasting temperature of the blends remained more or less the same. A higher peak viscosity was observed for wheat flour (2524 ± 26 mPa·s), presumably resulting from the fact that the highest starch content was obtained in WF compared to WWM and WRM. A similar observation was reported by Bae et al. [27]. During the holding period of the test, the viscosity dropped due to the melting of the crystalline regions of the starch granule, thereby causing a water flow into the granules [28]. The breakdown viscosity of WF was the highest (1647 ± 25 mPa·s) as a result of the high peak viscosity. This indicates a high degree of swelling of the starch granules and of amylose leaching during heating. The BD of WRM was the lowest, indicating that the starch granules in this matrix were most resistant to heating, resulting in a rather stable viscosity upon heating. Ragaee and Abdel-Aal [29] mentioned that the end-product quality of heat-treated cereal products is often positively correlated with peak viscosity. During cooling, the viscosity will increase again due to the re-alignment of mainly amylose, resulting in a gel structure [28]. The recrystallization of amylopectin will occur much later in the process, for example, in bread that is stored over a longer period of time (days or weeks) [30].

Setback (SB) is associated with retrogradation of starch and its ability to undergo syneresis. The total SB was the highest for WRM (1579 ± 63 mPa·s) and the lowest for WWM (1060 ± 31 mPa·s). The relative SB_t, rel_ (SB_t_/Final viscosity × 100%) is a better indication of retrogradation behavior, as it excludes potential interactions with the previous gelatinization behavior of starch and only gives an indication of the retrogradation rate of starch molecules in the gel [29]. SB_t, rel_ was the highest for WF (60.8%), followed by WWM:WF (58.3%) and the others (≤56.9%).

The starch pasting profiles showed that WRM gives the best pasting stability (highest value of HS). Furthermore, due to the presence of arabinoxylans, rye flour has a higher tendency to absorb water compared to wheat flour [31].

### 3.2. Crumb Core Temperatures

**Figure 2:**
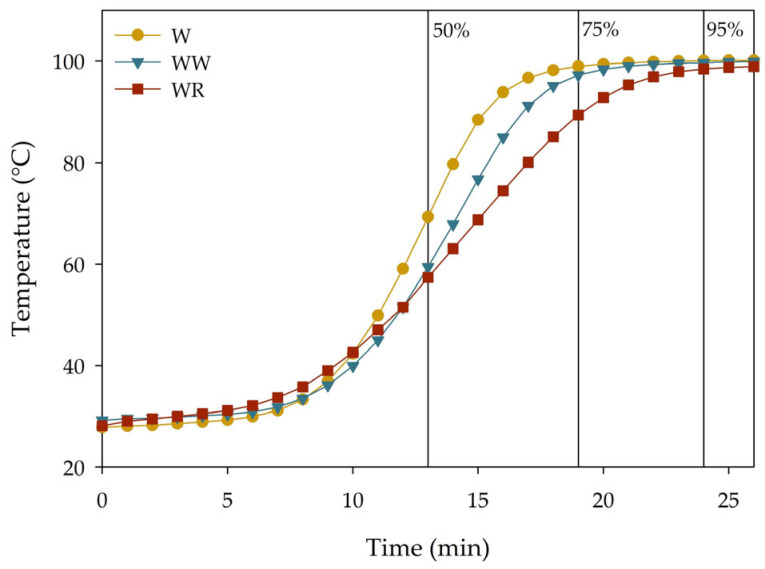
Core temperature of wheat (W), whole wheat (WW), and whole rye (WR) bread during baking. Vertical lines (50, 75, and 95%) indicate par-baking time.

Selected for this study were 50, 75, and 95% of the full baking time as the degrees of par-baking. The crumb core temperature was measured and is presented in Figure 2. The ideal crumb core temperature of bread is determined based on a combination of the full gelatinization of starch, protein coagulation, and desired bread texture and moisture content [32]. This temperature is dependent on the composition of the bread dough. For PB wheat bread, a final crumb core temperature of 93–98 °C is often reported [16,33,34,35]. Table 2 shows an overview of the crumb core temperatures reached at the end of the PB programs that were applied in this study. The crumb core temperature at 50% PB of WB and WWB did reach the pasting temperature of 57.3 and 59.3 °C, respectively, whereas this was not the case for WRB (pasting temperature: 59.7 °C).

Within this study, the maximal heating rate of WB was the highest (10.3 °C/min), followed by WWB (8.3 °C/min) and WRB (5.8 °C/min). The breads were all baked in the same oven and in the same baking pans; the volume of the breads after proofing did, however, slightly differ. Differences in bread volume and flour composition led to different heating rates. Patel, Waniska, and Seetharaman [36] found that the rate of bread firming was lower at slower heating rates. Although in this experiment, the heating rates of bread baking were not altered within the bread formulations (baking profile was stopped at 50, 75, and 95% of the complete baking time), it is interesting to acknowledge the effect of the heating rate on bread firming. Prior studies have noted the correlation between the heating rate and both an increased crumb firmness and faster staling kinetics [36,37,38,39]. Higher heating rates lead to increased amylose leaching and starch network formation, causing more starch recrystallization during bread storage, ultimately contributing to an accelerated crumb firming rate.

### 3.3. Bread Characterteristics

#### 3.3.1. The Influence of Composition on Par-Baked Bread

One of the research questions was whether a natural strategy for increased water retention during bread storage would be beneficial for extending bread quality and slowing down the staling process of bread. Two strategies were considered for increasing the water absorption in bread: lowering the degree of PB and enhancing wheat bread formulation with either whole wheat meal or whole rye meal. Whole wheat is known for its dietary fiber/bran content [27,40], whereas whole rye contains arabinoxylans and pentosans, enhancing the water absorption potential of bread [31,41,42]. A visual representation of the baked breads, which includes images of the par-baked and fully baked PB breads, as well as cut slices of fully baked PB breads, is presented in Figures S1 and S2 of the Supplementary Materials.

Looking at the crumb MC and bread weight (Table 3), both degrees of PB and bread formulation had only a minor impact on the absolute values. For instance, the MC of WRB at PB0-95% was 47.1 ± 0.2%, compared to 45.0 ± 0.1% and 45.0 ± 0.2% for WB and WWB, respectively. Similar values were found for the different degrees of PB, with a minor increase of max. 0.6% from 95 to 50% PB. This result is in accordance with the results of previous studies [6,10]. A varying degree of PB from 95 to 50% resulted in an increase in bread weight of max. 5–6% of the total weight at 95% PB. Interestingly, no additional water retention was observed for WWB, but a slightly increased MC was noted at the other stages of production (PB4, FB0, and FB2) for WWB compared to WB. This higher crumb moisture content for the composite breads is likely related to a larger amount of water in the formulation due to the presence of bran/fiber for WWB and the presence of arabinoxylans and pentosans in WRB [27,31,40,41,42].

Another finding emerging from the results (Table 3) was a weight loss of the loaf during the second baking phase (−3.51%, −3.41%, and −3.19% for WB, WWB, and WRB at 95% PB, similar for other degrees of PB), whereas no MC loss could be detected in the crumb. On the contrary, minor increases in crumb MC were found (+1.35%, +0.89%, and +0.42% for WB, WWB, and WRB at 95% PB, similar for other degrees of PB). This could be explained by the exclusion of water from the retrograded amylopectin network during the full-bake phase. During the staling of the PB breads, water had been incorporated into the starch crystalline structures [43,44]. Thus, it seems possible that the additional weight loss in the second baking phase can only be attributed to moisture loss in the crust, leading to crust formation finalization [7,10], as is evident in the lower L*-values for breads at FB2 (Table 3), indicating a darkening of the crust.

**Figure 3:**
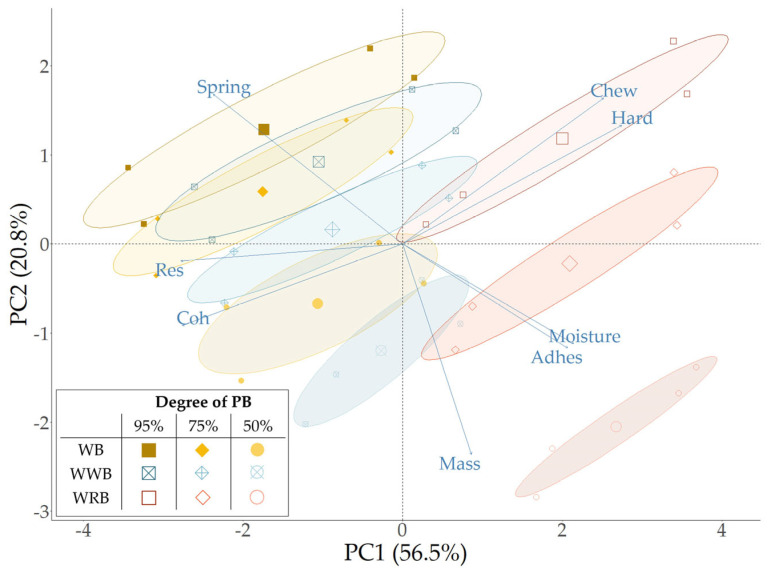
Principal component analysis of par-baked (PB) and fully baked PB breads based on composition—wheat (WB), whole wheat (WWB), and whole rye (WRB)—and degree of par-baking—95, 75, and 50% of complete baking time. Data for PB0, PB4, FB0, and FB2 are clustered by degree of PB and bread type.

In order to evaluate the effect of the composition on the quality properties of PB and fully baked PB breads, the attributes of PB0-95% were considered as reference for optimal quality and consumer acceptance. PB0-95% is characterized by high values of springiness, cohesion, and resilience, as well as low values of hardness, adhesiveness, and chewiness, representing the desired bread texture, which is associated with freshness. Although the absolute differences can seem rather small (Table 4), a principal component analysis (PCA) was performed to highlight the most relevant correlations between the degree of PB, flour composition, and bread characteristics. The two-dimensional PCA plot, comprising 77.3% of the total variance (PC 1: 56.5%; PC 2: 20.8%), is presented in Figure 3. Additional information on the PCA plot (e.g., correlations, scree plot) is presented in Figure S3 in the Supplementary Materials. This plot shows that the MC, adhesiveness, and springiness are most correlated with the degree of PB. In contrast, hardness, chewiness, resilience, and cohesiveness were not only correlated with the degree of PB, but also strongly dependent on time (e.g., PB0, PB4, FB0, FB2).

Whole rye bread, followed by whole wheat bread, showed the highest values for crumb hardness and chewiness (e.g., hardness values at 95%-PB0 for WRB: 1102 ± 57 g; for WWB: 416 ± 91 g; for WB: 284 ± 24 g), with increasing values for higher degrees of PB. This means that the hardness (firmness) was the highest for breads that were par-baked at 95%. The PB0 hardness values for WRB were 1102 ± 57 g, 925 ± 52 g, and 683 ± 11 g, respectively, for PB at 95%, 75%, and 50%. Similarly, Karaoglu [45] found that whole rye flour breads with a shorter initial baking time showed a higher softness value after cooled storage and full baking.

The degree of PB also influenced crumb springiness. A higher degree of PB resulted in a higher springiness. Whereas springiness is defined as the ratio of the height recovery of the crumb during the waiting period between the two compressions of the TPA [46], it is an important sensorial quality parameter for determining bread freshness. Wheat breads that were par-baked at 95% exhibited the overall highest values for springiness (0.94 ± 0.1, PB0 and FB0). For rye bread, springiness was most affected by the degree of PB at PB0. The springiness value decreased from 0.83 ± 0.02 at 95% to 0.66 ± 0.03 at 50% PB. At PB4, FB0, and FB2, the effects of the degree of PB were also present but resulted in smaller absolute differences. Furthermore, only a small correlation was found between crumb resilience and the degree of PB. Resilience is defined as the area under the curve after the first peak force is reached, divided by the area under the curve before that peak force is reached [46]. Resilience was strongly correlated with crumb cohesion. The lack of a strong correlation between the degree of PB and cohesion was due to the fact that the impact of the storage time on cohesion was far greater than the degree of PB. The effect of time will be discussed in the following section.

Enhancing wheat breads with whole meal causes unfavorable effects in terms of the desired bread quality attributes. The findings for WWB in this study align with previous results by Hemdane et al. [47] and Curti et al. [48] and can be attributed to fiber components affecting the starch–gluten–water interactions. For WRB, similar quality reductions were reported by Buksa et al. [42] and Bucsella et al. [49] and are, besides the level of fibers compared to the starch amount, also caused by starch type differences. In this study, that effect has also been observed in the pasting properties of WRM (Figure 1).

All these findings suggest that the strategy of naturally increasing water retention in bread through the composition does not prevent the staling process of PB bread. Moreover, the composition effect dominates the impact of the degree of PB on the quality properties. Consequently, for each bread type, the optimal degree of PB should be determined separately.

#### 3.3.2. The Influence of Storage Time and Degree of Par-Baking on Fully Baked Bread Quality

**Figure 4:**
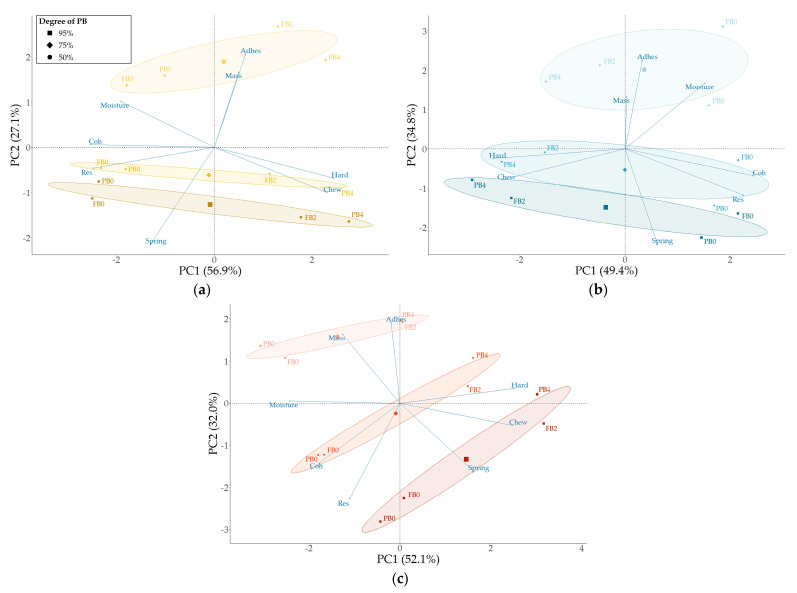
Principal component analysis of par-baked (PB) and fully baked PB breads based on composition—wheat ((**a**) yellow), whole wheat ((**b**) blue), and whole rye ((**c**) red)—and degree of par-baking—95, 75, and 50% of complete baking time (lighter shade indicates shorter baking times). Data for PB0, PB4, FB0, and FB2 are clustered by degree of PB.

The detailed PCA plots for the different bread types are shown in Figure 4 with the different times before and after full baking of the breads indicated as well (e.g., PB0, PB4, FB0, FB2). The analyses for wheat, whole wheat, and whole rye bread explain, respectively, 84.0%, 84.2%, and 84.1% of the variation. Figures S4–S6 in the Supplementary Materials present additional information on the PCA plots. Considering that bread is not always consumed immediately after baking, i.e., bread loaves versus baguette-type breads, the influence of the degree of par-baking and composition on the bread quality attributes as a function of time were assessed.

At FB0-95% and FB0-75%, no significant differences were observed for each of the bread types. Reducing the degree of PB to 50% resulted positively in decreased hardness and chewiness, but negatively impacted springiness, cohesion, and resilience. The crumb adhesion of WB was not influenced by the degree of PB, whereas for WWB and WRB, adhesion increased (Table 4). These findings would indicate that a degree of PB that is lower than 75% is not beneficial for reaching the optimal reference quality at FB0.

Over-time decreases in springiness, cohesion, and resilience, along with an increase in hardness, adhesiveness, and chewiness, are considered to result in a less desirable product. A reduction in the degree of PB from 95 to 50% resulted, at FB2, in less hardening, a lower increase in chewiness, and a smaller cohesion loss, but also promoted a larger loss of springiness and increased adhesion. Based on these results, a lower degree of PB would only be beneficial for crumb hardness, chewiness, and cohesion of bread as a function of storage time. Other authors [6,50,51] have reported that reducing the PB time was beneficial for reducing the staling rate. A shorter baking time resulted in a less extended starch network, leading to reduced retrogradation and a lower crumb firming rate.

These results indicate that the effect of the degree of PB differs for fresh bread (day 0) versus stale bread (day 2). Breads with consumption spread out over several days would benefit from a lower degree of PB regarding crumb hardness and cohesion, but this would inevitably also imply a reduction in crumb springiness and increase in adhesion. One could argue that both a 95% and 50% degree of PB will negatively affect bread sensorial quality when consumption is spread out over time, making 75% PB the middle ground. Based on the results of the core temperature measurement, this matches with reaching core temperatures of, respectively, 99.2 °C, 97.9 °C, and 91.3 °C for WB, WWB, and WRB, respectively, along with corresponding heating rates of 10.3 °C/min, 8.3 °C/min, and 5.8 °C/min for this study. As previously mentioned, a reduced heating rate could also contribute to reduced staling kinetics.

## 4. Conclusions

Within the industry and academia, par-baking programs are often defined based on reaching certain core temperatures, expressed as a shortened baking time or as a percentage of the full baking time. Therefore, this study aimed at redefining the scope of par-baking of (composite) wheat breads, supported with data on core temperature, baking programs, and bread quality characteristics. Furthermore, this study investigated the potential of increasing the bread’s freshness through natural water retention in bread.

For par-baked breads consumed on day 0 (immediately after full baking), the degree of PB did not influence end-product quality. However, with storage time, the quality was strongly impacted in terms of the degree of PB. Setting the degree of PB based solely on the core temperature is insufficient in light of the results of this study. For example, a core temperature of 95 °C can be insufficient for several bread formulations, which results in a lower degree of PB (<75%) and a negative impact on the bread quality: lower springiness and higher adhesion. Also, the heating rate during the baking phases needs consideration to reduce staling mechanisms. Overall, this study was able to highlight the disadvantages of very short (50%) and very long (95% of total baking time) PB programs in terms of the quality of fully baked par-baked wheat, whole wheat, and whole rye bread (consumed on day 2). Therefore, a PB program in the medium range (75% of total baking time) could be considered. However, in an industrial setting, many additional factors, e.g., the convenience for hot points (i.e., lower full baking times in the shops) and the risk of puncture of packaging material (when crust is harder), need to be considered, leaving room for optimization. Additionally, a lower degree of PB also results in a higher crumb moisture content and water activity (a_w_) of bread, which makes the products more susceptible to microbiological spoilage. In addition to bread quality aspects, the general aspects of production cost and sustainability should be considered.

Future studies can further optimize the degree of PB for different bread formulations with respect to the differences in heating rates for small–large breads, baguettes–pan breads–plate breads, plain–complex formulations, etc. Based on a sensorial evaluation of these bread formulations, the most relevant physico-chemical parameters should be highlighted to optimize the PB process for these diverse formulations.

## Figures and Tables

**Table 1:** Farinograph water absorption (%, on flour weight basis, corrected for 14% moisture content) and dough moisture content (%) for wheat flour (W) and 50:50 blends consisting of WWM:WF (WW) and WRM:WF (WR).

	Farinograph Water Absorption (%)	Dough Moisture Content (%)
W	58.5	44.9
WW	58.3	45.0
WR	65.0	46.7

**Table 2:** Final bread core temperatures (°C) of wheat (W), whole wheat (WW), and whole rye (WR) breads that were 50, 75, and 95% par-baked (*n* = 2).

	Final Bread Core Temperatures (°C)		
	50% PB	75% PB	95% PB
W	69.3	99.2	100
WW	59.5	97.9	99.8
WR	57.4	91.3	98.7

**Table 3:** Moisture content (MC, %, *n* = 6), weight (m, g, *n* = 24), and L*, a*, b* (*n* = 16) color values for wheat (W), whole wheat (WW), and whole rye (WR) breads, measured at PB0, PB4, FB0, and FB2, with 95, 75, and 50% as initial degrees of PB.

		PB	MC (%)	m (g)	L*	a*	b*
W	PB0	95	45.0 ± 0.1 ^ab^	346.0 ± 2.1 ^a^	52.9 ± 4.2 ^a^	16.7 ± 1.2 ^a^	34.2 ± 3.5 ^ac^
		75	44.7 ± 0.4 ^a^	354.1 ± 1.7 ^ab^	54.4 ± 4.7 ^a^	16.1 ± 1.3 ^ac^	35.0 ± 3.2 ^ac^
		50	44.8 ± 0.4 ^a^	364.5 ± 1.3 ^b^	59.9 ± 2.9 ^a^	14.2 ± 1.1 ^ab^	36.8 ± 1.0 ^a^
	PB4	95	44.4 ± 0.4 ^a^	347.1 ± 1.4 ^a^	-	-	-
		75	44.3 ± 0.4 ^a^	354.8 ± 1.1 ^ab^	-	-	-
		50	44.7 ± 0.3 ^a^	364.7 ± 1.3 ^b^	-	-	-
	FB0	95	45.0 ± 0.4 ^a^	334.9 ± 1.2 ^a^	42.1 ± 3.3 ^bc^	14.1 ± 0.7 ^ab^	24.4 ± 1.9 ^be^
		75	45.0 ± 0.3 ^ab^	341.7 ± 1.4 ^a^	45.8 ± 5.5 ^abc^	13.9 ± 0.8 ^ab^	26.7 ± 4.2 ^bcde^
		50	45.2 ± 0.4 ^ab^	350.3 ± 2.0 ^ab^	48.2 ± 3.8 ^abc^	14.2 ± 0.5 ^ab^	29.0 ± 2.6 ^abd^
	FB2	95	44.7 ± 0.4 ^a^	334.5 ± 0.8 ^a^	-	-	-
		75	44.8 ± 0.3 ^a^	342.2 ± 1.2 ^a^	-	-	-
		50	45.0 ± 0.4 ^ab^	350.5 ± 1.6 ^ab^	-	-	-
WW	PB0	95	45.0 ± 0.2 ^ab^	350.0 ± 1.2 ^ab^	48.4 ± 2.7 ^ab^	15.5 ± 0.7 ^ac^	29.5 ± 2.1 ^abd^
		75	45.1 ± 0.2 ^ab^	356.8 ± 1.0 ^b^	50.3 ± 2.2 ^a^	15.3 ± 0.9 ^ab^	30.2 ± 2.3 ^abd^
		50	45.3 ± 0.2 ^ab^	366.7 ± 0.9 ^b^	53.0 ± 2.4 ^a^	14.6 ± 1.1 ^ab^	31.3 ± 1.4 ^ad^
	PB4	95	44.9 ± 0.2 ^a^	348.9 ± 1.1 ^a^	-	-	-
		75	45.0 ± 0.2 ^ab^	356.0 ± 1.1 ^b^	-	-	-
		50	45.2 ± 0.2 ^ab^	366.4 ± 1.1 ^b^	-	-	-
	FB0	95	45.3 ± 0.2 ^ab^	337.0 ± 1.3 ^a^	41.7 ± 2.6 ^c^	13.0 ± 0.7 ^b^	23.0 ± 2.0 ^be^
		75	45.5 ± 0.2 ^ab^	344.5 ± 1.4 ^a^	43.3 ± 2.4 ^bc^	12.9 ± 0.8 ^b^	23.5 ± 2.6 ^be^
		50	45.7 ± 0.2 ^ab^	353.4 ± 1.3 ^ab^	45.1 ± 1.9 ^abc^	13.2 ± 0.7 ^b^	25.0 ± 1.8 ^bde^
	FB2	95	45.0 ± 0.1 ^ab^	337.6 ± 1.7 ^a^	-	-	-
		75	45.3 ± 0.2 ^ab^	344.7 ± 1.8 ^a^	-	-	-
		50	45.4 ± 0.1 ^ab^	353.3 ± 1.9 ^ab^	-	-	-
WR	PB0	95	47.1 ± 0.2 ^b^	350.9 ± 1.0 ^a^	45.0 ± 2.1 ^abc^	14.8 ± 1.0 ^ab^	27.5 ± 2.0 ^bde^
		75	47.2 ± 0.2 ^b^	358.5 ± 1.0 ^b^	47.4 ± 2.1 ^abc^	14.6 ± 0.6 ^ab^	28.9 ± 1.6 ^abcde^
		50	47.4 ± 0.2 ^b^	367.7 ± 0.8 ^b^	50.2 ± 1.8 ^abc^	13.6 ± 1.1 ^ab^	29.2 ± 1.2 ^abcd^
	PB4	95	46.7 ± 0.0 ^b^	350.9 ± 0.9 ^a^	-	-	-
		75	46.8 ± 0.1 ^b^	358.5 ± 1.1 ^b^	-	-	-
		50	47.0 ± 0.2 ^b^	367.6 ± 0.9 ^b^	-	-	-
	FB0	95	46.9 ± 0.2 ^b^	339.7 ± 1.2 ^a^	39.0 ± 2.6 ^c^	13.2 ± 0.4 ^b^	21.5 ± 1.8 ^e^
		75	47.3 ± 0.4 ^b^	348.0 ± 1.1 ^a^	42.5 ± 1.4 ^bc^	13.5 ± 0.9 ^bc^	24.3 ± 1.1 ^bde^
		50	47.5 ± 0.2 ^b^	355.9 ± 0.9 ^b^	47.0 ± 1.9 ^abc^	13.2 ± 0.6 ^b^	26.5 ± 1.2 ^abcde^
	FB2	95	46.8 ± 0.2 ^b^	339.6 ± 1.6 ^a^	-	-	-
		75	47.0 ± 0.9 ^b^	348.3 ± 1.0 ^ab^	-	-	-
		50	47.0 ± 0.3 ^b^	355.9 ± 1.1 ^b^	-	-	-

a–e: Values in the same column followed by different superscripts are significantly different (*p* < 0.05, with Bonferroni correction).

**Table 4:** Bread crumb texture: hardness (g), adhesiveness (g), springiness, cohesiveness, chewiness, and resilience for wheat (W), whole wheat (WW), and whole rye (WR) breads, measured at PB0, PB4, FB0, and FB2, with 95, 75, and 50% as initial degrees of PB (*n* = 8).

		PB	Hard. (g)	Adhes. (g)	Spring. (-)	Coh. (-)	Chew. (g)	Res. (-)
W	PB0	95	284 ± 24 ^a^	−4 ± 3 ^a^	0.94 ± 0.01 ^ab^	0.83 ± 0.01 ^a^	222 ± 18 ^a^	0.47 ± 0.01 ^a^
		75	260 ± 29 ^a^	−3 ± 3 ^a^	0.92 ± 0.01 ^ab^	0.82 ± 0.01 ^a^	205 ± 29 ^a^	0.46 ± 0.01 ^a^
		50	223 ± 31 ^bc^	−5 ± 2 ^a^	0.84 ± 0.03 ^abcd^	0.76 ± 0.04 ^a^	140 ± 12 ^a^	0.37 ± 0.04 ^a^
	PB4	95	1125 ± 97 ^bc^	−3 ± 2 ^a^	0.90 ± 0.02 ^abc^	0.51 ± 0.01 ^b^	523 ± 63 ^b^	0.21 ± 0.02 ^bc^
		75	1004 ± 67 ^abc^	−4 ± 2 ^a^	0.88 ± 0.02 ^abcd^	0.58 ± 0.03 ^bc^	509 ± 47 ^b^	0.24 ± 0.02 ^bc^
		50	831 ± 42 ^a^	−11 ± 4 ^a^	0.81 ± 0.03 ^abcd^	0.57 ± 0.02 ^bc^	385 ± 15 ^ab^	0.21 ± 0.01 ^bc^
	FB0	95	293 ± 50 ^a^	−5 ± 2 ^a^	0.94 ± 0.01 ^a^	0.84 ± 0.02 ^a^	229 ± 34 ^a^	0.49 ± 0.02 ^a^
		75	260 ± 27 ^a^	−4 ± 1 ^a^	0.92 ± 0.01 ^ab^	0.81 ± 0.02 ^a^	194 ± 21 ^a^	0.45 ± 0.02 ^a^
		50	213 ± 17 ^a^	−6 ± 2 ^a^	0.85 ± 0.03 ^abcd^	0.77 ± 0.03 ^a^	138 ± 5 ^a^	0.37 ± 0.04 ^ab^
	FB2	95	995 ± 111 ^bc^	−4 ± 2 ^a^	0.88 ± 0.02 ^ab^	0.58 ± 0.03 ^bc^	513 ± 71 ^b^	0.26 ± 0.02 ^bc^
		75	802 ± 76 ^abc^	−7 ± 4 ^a^	0.89 ± 0.03 ^ab^	0.61 ± 0.02 ^ab^	437 ± 34 ^ab^	0.26 ± 0.02 ^abc^
		50	678 ± 67 ^ab^	−19 ± 3 ^a^	0.80 ± 0.03 ^bd^	0.63 ± 0.03 ^ab^	340 ± 23 ^ab^	0.25 ± 0.03 ^bc^
WW	PB0	95	416 ± 91 ^ab^	−2 ± 0 ^a^	0.90 ± 0.02 ^ab^	0.79 ± 0.02 ^ac^	294 ± 58 ^a^	0.42 ± 0.02 ^a^
		75	348 ± 8 ^ab^	−2 ± 1 ^a^	0.88 ± 0.03 ^abd^	0.78 ± 0.02 ^ac^	237 ± 20 ^a^	0.41 ± 0.02 ^a^
		50	271 ± 17 ^a^	−5 ± 1 ^a^	0.78 ± 0.03 ^cd^	0.73 ± 0.03 ^a^	154 ± 14 ^a^	0.33 ± 0.03 ^ab^
	PB4	95	1174 ± 88 ^bc^	−5 ± 4 ^a^	0.84 ± 0.04 ^abcd^	0.51 ± 0.02 ^b^	501 ± 60 ^b^	0.20 ± 0.02 ^bc^
		75	1060 ± 124 ^bc^	−4 ± 3 ^a^	0.83 ± 0.04 ^abcd^	0.54 ± 0.02 ^b^	470 ± 41 ^b^	0.21 ± 0.02 ^bc^
		50	864 ± 99 ^abc^	−9 ± 5 ^a^	0.77 ± 0.04 ^cd^	0.56 ± 0.02 ^bc^	370 ± 61 ^ab^	0.20 ± 0.01 ^bc^
	FB0	95	368 ± 47 ^ab^	−5 ± 2 ^a^	0.90 ± 0.02 ^abc^	0.79 ± 0.02 ^ac^	263 ± 28 ^a^	0.43 ± 0.02 ^a^
		75	364 ± 25 ^ab^	−8 ± 3 ^a^	0.87 ± 0.04 ^abcd^	0.78 ± 0.01 ^ac^	244 ± 14 ^a^	0.40 ± 0.02 ^a^
		50	285 ± 30 ^a^	−15 ± 5 ^a^	0.75 ± 0.04 ^d^	0.70 ± 0.04 ^ab^	149 ± 3 ^a^	0.29 ± 0.04 ^ab^
	FB2	95	1064 ± 80 ^bc^	−4 ± 1 ^a^	0.84 ± 0.03 ^abcd^	0.57 ± 0.03 ^bc^	510 ± 43 ^b^	0.24 ± 0.01 ^bc^
		75	997 ± 45 ^bc^	−5 ± 2 ^a^	0.81 ± 0.03 ^abcd^	0.60 ± 0.02 ^b^	484 ± 29 ^b^	0.24 ± 0.02 ^bc^
		50	724 ± 51 ^abc^	−12 ± 4 ^a^	0.77 ± 0.05 ^cd^	0.60 ± 0.02 ^b^	334 ± 33 ^ab^	0.22 ± 0.02 ^bc^
WR	PB0	95	1102 ± 57 ^bc^	−12 ± 4 ^a^	0.83 ± 0.02 ^abcd^	0.71 ± 0.01 ^a^	653 ± 52 ^b^	0.38 ± 0.01 ^ab^
		75	925 ± 52 ^abc^	−20 ± 4 ^a^	0.75 ± 0.02 ^d^	0.69 ± 0.02 ^a^	472 ± 37 ^b^	0.33 ± 0.02 ^ab^
		50	683 ± 116 ^ab^	−68 ± 16 ^b^	0.66 ± 0.03 ^d^	0.60 ± 0.03 ^ab^	276 ± 71 ^a^	0.23 ± 0.03 ^bc^
	PB4	95	2406 ± 161 ^c^	−44 ± 7 ^b^	0.79 ± 0.05 ^cde^	0.43 ± 0.01 ^b^	812 ± 55 ^b^	0.16 ± 0.01 ^c^
		75	1996 ± 186 ^c^	−79 ± 26 ^b^	0.75 ± 0.04 ^d^	0.45 ± 0.02 ^b^	676 ± 92 ^b^	0.16 ± 0.01 ^c^
		50	1420 ± 208 ^abc^	−238 ± 33 ^c^	0.76 ± 0.04 ^d^	0.54 ± 0.03 ^b^	578 ± 109 ^b^	0.15 ± 0.01 ^c^
	FB0	95	1159 ± 76 ^bc^	−57 ± 28 ^b^	0.78 ± 0.04 ^cde^	0.68 ± 0.02 ^abc^	618 ± 60 ^b^	0.34 ± 0.02 ^ab^
		75	926 ± 87 ^abc^	−51 ± 12 ^b^	0.74 ± 0.04 ^d^	0.67 ± 0.02 ^ac^	464 ± 61 ^b^	0.30 ± 0.02 ^abc^
		50	646 ± 105 ^ab^	−187 ± 59 ^bc^	0.72 ± 0.04 ^d^	0.61 ± 0.05 ^ac^	288 ± 70 ^a^	0.20 ± 0.03 ^bc^
	FB2	95	2364 ± 262 ^c^	−66 ± 22 ^b^	0.79 ± 0.03 ^cde^	0.46 ± 0.01 ^b^	872 ± 126 ^b^	0.18 ± 0.01 ^c^
		75	2042 ± 152 ^c^	−98 ± 32 ^b^	0.75 ± 0.05 ^d^	0.49 ± 0.02 ^b^	749 ± 51 ^b^	0.18 ± 0.01 ^c^
		50	1446 ± 214 ^bc^	−302 ± 33 ^c^	0.73 ± 0.07 ^d^	0.54 ± 0.03 ^b^	579 ± 120 ^b^	0.16 ± 0.02 ^c^

a–e: Values in the same column followed by different superscripts are significantly different (*p* < 0.05, with Bonferroni correction).

## Data Availability

The data presented in this study are available in the article and Supplementary Material.

## Supplementary Materials

The following supporting information can be downloaded at https://www.mdpi.com/article/10.3390/foods13020224/s1: Table S1: Pasting parameters (initial viscosity (IV), pasting temperature (PaT), peak viscosity (PeV), peak temperature (PeT), holding strength (HS), breakdown (BD), final viscosity (FV), setback from peak (SBp), and total setback (SBt)) of wheat flour (WF), whole wheat meal (WWM), whole rye meal (WRM) (n = 6), and 50:50 blends of WWM:WF and WRM:WF (n = 3); Figure S1: Par-baked (left) and fully baked (right) wheat, whole wheat, and whole rye breads, previously par-baked at (a) 95, (b) 75, and (c) 50% of the normal baking time; Figure S2: Bread slices of fully baked wheat, whole wheat, and whole rye breads previously par-baked at (a) 95, (b) 75, and (c) 50% of the normal baking time; Figure S3: Correlation plot (a), scree plot (b), and visual representation of the importance of the individual factors in the dimensions of the PCA plot (c) for data for all bread types combined (W, WW, and WR); Figure S4: Correlation plot (a), scree plot (b), and visual representation of the importance of the individual factors in the dimensions of the PCA plot (c) for data for wheat bread; Figure S5: Correlation plot (a), scree plot (b), and visual representation of the importance of the individual factors in the dimensions of the PCA plot (c) for data for whole wheat bread; Figure S6: Correlation plot (a), scree plot (b), and visual representation of the importance of the individual factors in the dimensions of the PCA plot (c) for data for whole rye bread.
