# Supplementary material for: An Impact Assessment of Par-Baking and Storage on the Quality of Wheat, Whole Wheat, and Whole Rye Breads
Source: Foods. 2024 Jan 10;13(2):224. doi: 10.3390/foods13020224 (PMC10814006; doi:10.3390/foods13020224)
Supplement: Supplementary file 1 [file foods-13-00224-s001.zip › foods-2810651-supplementary.pdf]

foods

Supplementary Materials

Table S1. Pasting parameters [initial viscosity (IV), pasting temperature (PaT), peak viscosity (PeV), peak temperature (PeT), holding strength (HS), breakdown (BD), final viscosity (FV), setback from peak (SBp), and total setback (SBt)] of wheat flour (WF), whole wheat meal (WWM), whole rye meal (WRM) ($n = 6$), and 50:50 blends of WWM:WF and WRM:WF ($n = 3$).

Pasting Parameter	WF	WWM:WF	WWM	WRM:WF	WRM
IV (mPa·s)	2.8 ± 0.0 ^{a}	5.6 ± 2.2 ^{ab}	30.4 ± 15.9 ^{ab}	21.5 ± 16.8 ^{b}	67.0 ± 26.0 ^{b}
PaT (°C)	57.6 ± 0.8 ^{a}	59.3 ± 0.1 ^{a}	73.1 ± 6.7 ^{b}	59.7 ± 2.2 ^{ab}	60.4 ± 3.4 ^{ab}
PeV (mPa·s)	2524 ± 26 ^{a}	1833 ± 84 ^{ab}	1231 ± 74 ^{b}	1991 ± 66 ^{ab}	1541 ± 86 ^{b}
PeT (°C)	92.0 ± 0.1 ^{a}	92.0 ± 0.2 ^{a}	92.2 ± 0.4 ^{a}	91.5 ± 0.3 ^{a}	91.9 ± 0.3 ^{a}
HS (mPa·s)	877 ± 5 ^{ab}	884 ± 45 ^{ab}	833 ± 47 ^{b}	997 ± 51 ^{a}	1203 ± 95 ^{a}
BD (mPa·s)	1647 ± 25 ^{a}	950 ± 50 ^{ab}	398 ± 58 ^{b}	994 ± 37 ^{ab}	338 ± 28 ^{b}
FV (mPa·s)	2232 ± 32 ^{ab}	2122 ± 109 ^{a}	1893 ± 94 ^{a}	2314 ± 70 ^{ab}	2782 ± 135 ^{b}
SBp (mPa·s)	-291 ± 9 ^{a}	288 ± 56 ^{ab}	662 ± 30 ^{a}	323 ± 40 ^{ab}	1241 ± 90 ^{a}
SBt (mPa·s)	1356 ± 32 ^{ab}	1238 ± 67 ^{ab}	1060 ± 31 ^{a}	1317 ± 39 ^{ab}	1579 ± 63 ^{b}

a-b: Values in the same row followed by different superscripts are significantly different ($p < 0.05$).

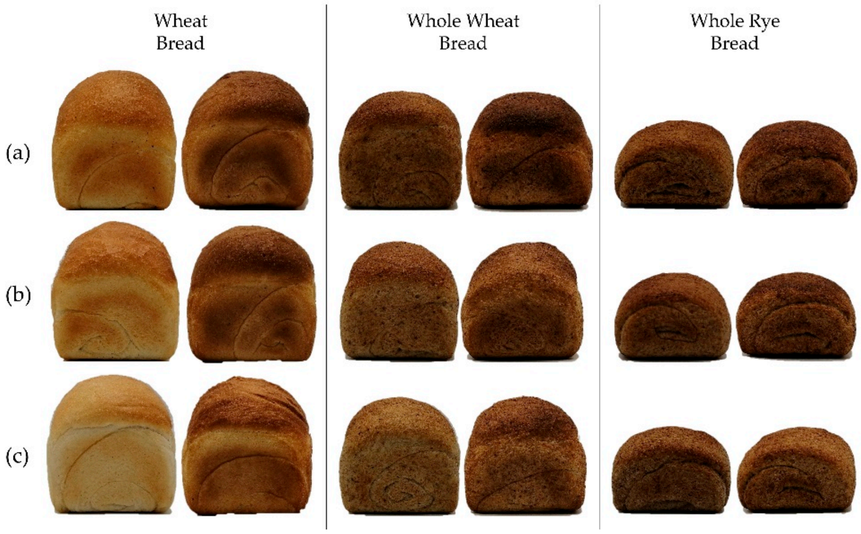

Figure S1: Par-baked (left) and full-baked (right) wheat, whole wheat, and whole rye breads previously par baked at a) 95, b) 75 and c) 50% of the normal baking time.

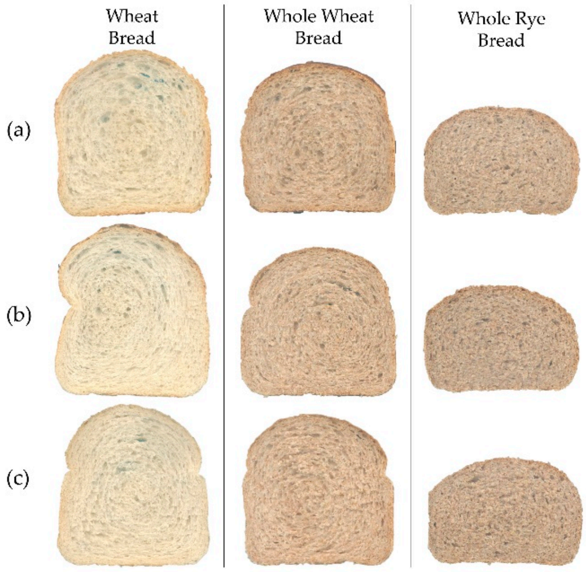

Figure S2: Bread slices of fully baked wheat, whole wheat, and whole rye breads previously par-baked at a) 95, b) 75 and c) 50% of the normal baking time.

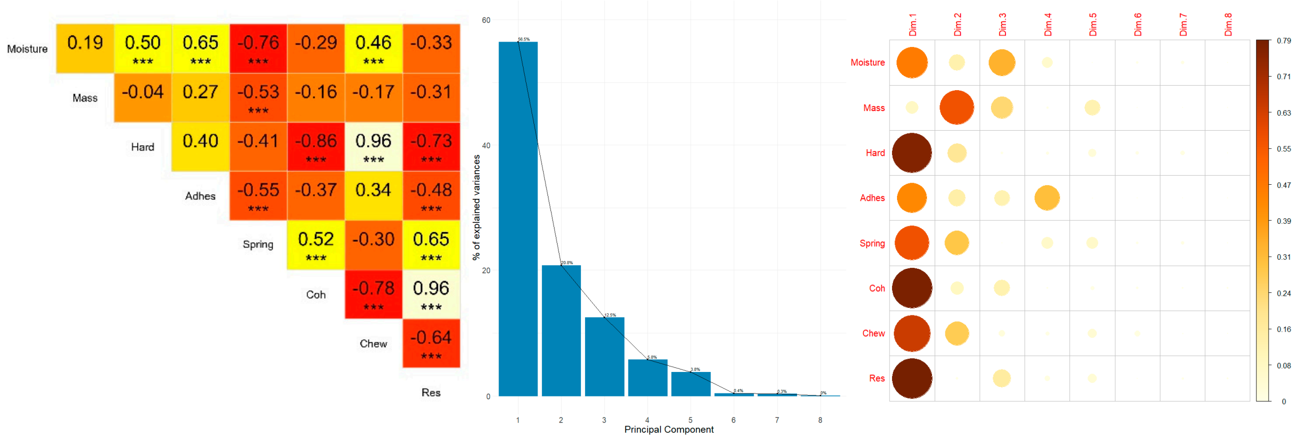

(a) (b) (c)

Figure S3: Correlation plot (a), scree plot (b) and visual representation of the importance of the individual factors in the dimensions of the PCA plot (c) – data of all bread types combined (W, WW and WR).

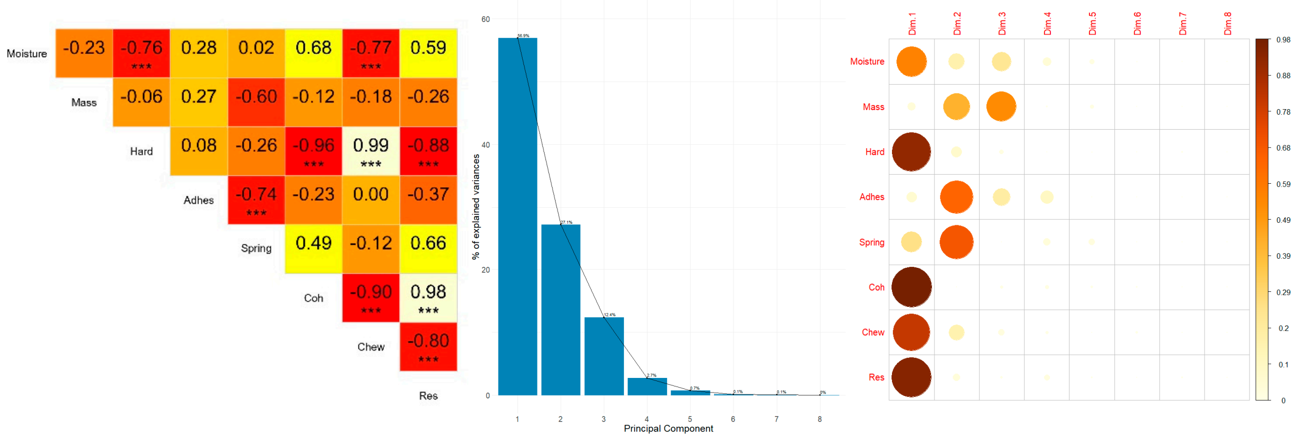

(a) (b) (c)

Figure S4: Correlation plot (a), scree plot (b) and visual representation of the importance of the individual factors in the dimensions of the PCA plot (c) – data of wheat bread.

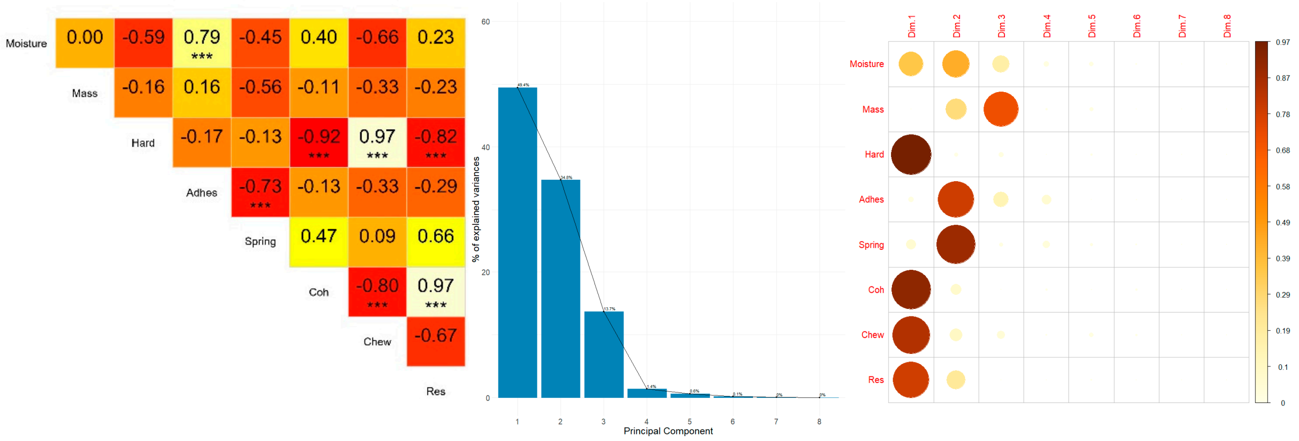

(a) (b) (c)

Figure S5: Correlation plot (a), scree plot (b) and visual representation of the importance of the individual factors in the dimensions of the PCA plot (c) – data of whole wheat bread.

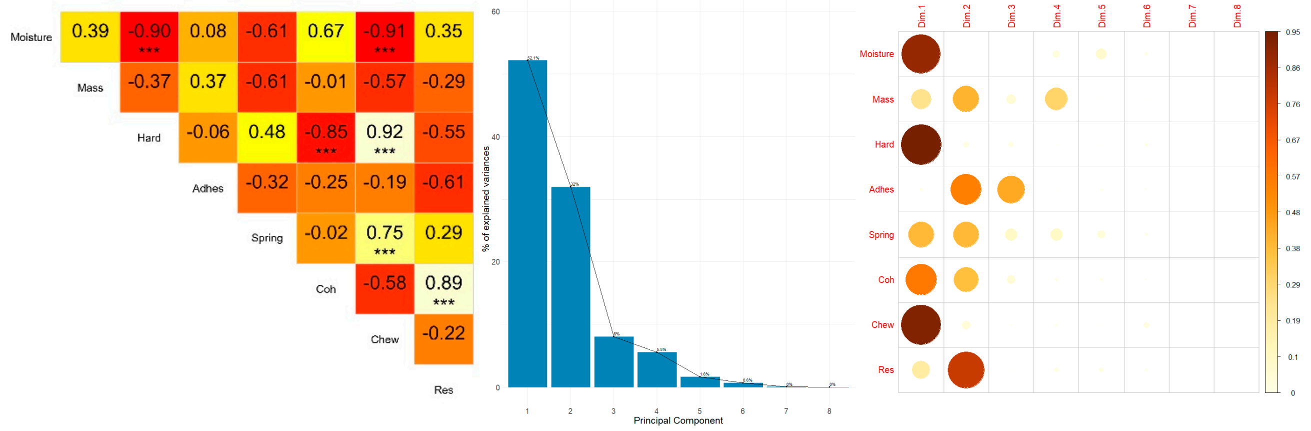

(a) (b) (c)

Figure S6: Correlation plot (a), scree plot (b) and visual representation of the importance of the individual factors in the dimensions of the PCA plot (c) – data of whole rye bread.
